# What are the recognized species of the genus *Listeria*?

**DOI:** 10.1099/acmi.0.000153

**Published:** 2020-07-21

**Authors:** Ogueri Nwaiwu

**Affiliations:** ^1^​ School of Biosciences, Sutton Bonington Campus, University of Nottingham, Sutton Bonington LE12 5RD, UK

**Keywords:** *Listeria* species, bacteria, taxonomy

*Listeria* is an important food-borne pathogen that affects the elderly, pregnant women, neonates and immune-compromised persons. In the last decade, there has been an increase in identification of new species from diverse sources, but *Listeria monocytogenes* remains the main pathogenic species of the genus. In recent times, investigators have reported different numbers of species for the genus; hence, it is important to clarify the number of species available now. A recent article [1] reported that the genus *Listeria* comprises up to 21 species. The authors relied on the information in the List of Prokaryotic Names with Standing in Nomenclature at the ‘Bacterio.net’ website (http://www.bacterio.net/listeria.html) to conclude 21 species. A closer perusal of the information on this site shows that some species taxonomy details were revised many years ago. The most pathogenic, *L. monocytogenes *, was originally described in 1926, but assigned the name in 1940 [2]. Although 22 species are listed, 2 species, namely *Listeria denitrificans* and *Listeria murrayi *, are shown to be synonyms. The species *L. denitrificans* was transferred to a new genus and renamed *Jonesia denitrificans* in 1987 [3] and has since been confirmed by two recent emendations [4, 5]. It was later found that high-level similarities exist between *Listeria grayi and L. murrayi* when a re-evaluation was carried out by Rocourt *et al*. [6] using several methods that included DNA–DNA hybridization, multilocus enzyme electrophoresis and rRNA restriction fragment length polymorphism. Hence, both were merged to a single species, *L. grayi *, as a matter of priority. The emended strain can be obtained using the designation ATCC 25401 [7]. The 2oth species described is *Listeria thailandensis* by Leclercq *et al*. [8] and they acknowledged 19 species existed before they described the new species. This is the last entry made in the *International Journal of Systematic and Evolutionary Microbiology*, the leading forum for the publication of novel microbial taxa and the International Committee on Systematics of Prokaryotes (ICSP) official journal record for prokaryotic names. The 20 species reviewed (Table 1) are the most up to date count of the number of *Listeria* species and not 21.

**Table 1.** The species of the genus *Listeria
*

No.	Species	Year	Reference
1	* L. thailandensis *	2019	[8]
2	* L. costaricensis *	2018	[9]
3	* L. goaensis *	2018	[10]
4	* L. newyorkensis *	2015	[11]
5	* L. booriae *	2015	[11]
6	* L. riparia *	2014	[12]
7	* L. grandensis *	2014	[12]
8	* L. floridensis *	2014	[12]
9	* L. cornellensis *	2014	[12]
10	* L. aquatica *	2014	[12]
11	* L. weihenstephanensis *	2013	[13]
12	* L. fleischmannii *	2013	[14]
13	* L. rocourtiae *	2010	[15]
14	* L. marthii *	2010	[16]
15	* L. grayi *	1992	[6]
16	* L. ivanovii *	1984	[17]
17	* L. welshimeri *	1983	[18]
18	* L. seeligeri *	1983	[18]
19	* L. innocua *	1983	[19]
20	* L. monocytogenes *	1940	[2]
